# Flipped Classroom: Improved team performance during resuscitation training through interactive pre-course content – a cluster-randomised controlled study

**DOI:** 10.1186/s12909-024-05438-7

**Published:** 2024-04-26

**Authors:** Hendrik Ohlenburg, Philip-Helge Arnemann, Michael Hessler, Dennis Görlich, Alexander Zarbock, Hendrik Friederichs

**Affiliations:** 1 Institute of Education and Student Affairs, Studienhospital Münster, University of Münster, 48149 Münster, Germany; 2 Department of Anaesthesiology, Intensive Care and Pain Medicine, Münster University Hospital, Münster, Germany; 3 Institute of Biostatistics and Clinical Research, University of Münster, Münster, Germany; 4 Medical Education Research Group, Medical School OWL, Bielefeld University, Bielefeld, Germany

**Keywords:** Flipped classroom, Resuscitation, Non-technical skills, Team training

## Abstract

**Background:**

Resuscitation is a team effort, and it is increasingly acknowledged that team cooperation requires training. Staff shortages in many healthcare systems worldwide, as well as recent pandemic restrictions, limit opportunities for collaborative team training. To address this challenge, a learner-centred approach known as flipped learning has been successfully implemented. This model comprises self-directed, asynchronous pre-course learning, followed by knowledge application and skill training during in-class sessions. The existing evidence supports the effectiveness of this approach for the acquisition of cognitive skills, but it is uncertain whether the flipped classroom model is suitable for the acquisition of team skills. The objective of this study was to determine if a flipped classroom approach, with an online workshop prior to an instructor-led course could improve team performance and key resuscitation variables during classroom training.

**Methods:**

A single-centre, cluster-randomised, rater-blinded study was conducted on 114 final year medical students at a University Hospital in Germany. The study randomly assigned students to either the intervention or control group using a computer script. Each team, regardless of group, performed two advanced life support (ALS) scenarios on a simulator. The two groups differed in the order in which they completed the flipped e-learning curriculum. The intervention group started with the e-learning component, and the control group started with an ALS scenario.

Simulators were used for recording and analysing resuscitation performance indicators, while professionals assessed team performance as a primary outcome.

**Results:**

The analysis was conducted on the data of 96 participants in 21 teams, comprising of 11 intervention groups and 10 control groups. The intervention teams achieved higher team performance ratings during the first scenario compared to the control teams (Estimated marginal mean of global rating: 7.5 vs 5.6, *p* < 0.01; performance score: 4.4 vs 3.8, *p* < 0.05; global score: 4.4 vs 3.7, *p* < 0.001). However, these differences were not observed in the second scenario, where both study groups had used the e-learning tool.

**Conclusion:**

Flipped classroom approaches using learner-paced e-learning prior to hands-on training can improve team performance.

**Trial registration:**

German Clinical Trials Register (https://drks.de/search/de/trial/DRKS00013096).

## Background

Cardiac arrest is a significant healthcare burden with socio-economic implications [1]. Sudden cardiac arrests account for 15–20% of all natural adult deaths in the USA and Western Europe [2]. Surviving a cardiac arrest is possible, when treated instantly, but the outcome depends on the weakest link in the *chain of survival* [3]. Efforts have been made to promote early recognition of cardiac arrest and the application of basic life support by bystanders. Although there may be deficits in the early phase of resuscitation [4], there is still room for improvement in later phases.

During the later phases of resuscitation, there is a high demand for resources and a heavy workload for the multidisciplinary team involved. The team must work together seamlessly and efficiently to achieve the best outcome for the patient. However, studies have shown that there is a lack of basic technical and non-technical skills among resuscitation teams during CPR [5–7]. Standardised course concepts aim to improve this part of the chain of survival. Effective team training has been shown to reduce medical errors and patient mortality [8]. Improved team performance is associated with better resuscitation outcomes. Cooper and colleagues found that team structure correlates highly with the dynamism and accuracy of measures during resuscitation [9]. Experimental studies have demonstrated that team structure and leadership can significantly impact performance [10]. Additionally, preformed teams have been shown to experience less hands-off time [11].

Several educational strategies have been developed to train resuscitation teams, each with its own advantages and disadvantages [12]. However, irrespective of the particular approach used, regular training is essential to maintain high-quality standards [13]. This poses a challenge for educational and training experts, as well as workforce management, due to the need for ongoing training, limited resources, and a shortage of healthcare workers [14].

One pedagogical strategy that can be employed is the flipped classroom [15, 16] (FC), which reverses the typical in-class learning and homework elements of a course. This learner-centered approach enables students to actively and independently acquire basic knowledge, which they can then apply and reflect upon in instructor-led in-class sessions. Knowledge can be acquired from a variety of sources, including books, textual e-learning materials, audio or video recordings, and pre-recorded lectures. According to Bloom's taxonomy [17], during the instructor-led in-class phase, higher-order cognitive processes follow lower-order cognitive processes during homework.

FC is not standardised, and different approaches are not directly comparable. According to a general definition, FC is the opposite of traditional classroom [18]. Scientific evidence on FC is consistently inconclusive. However, several studies have shown positive effects of flipped-classroom learning on cognitive and psychomotor skill acquisition [15, 19, 20]. Yoosoof et al. [21] demonstrated a significant improvement in certain domains of newborn resuscitation training for medical students through the use of elaborated preparation materials. The effectiveness of a FC approach depends on its implementation rather than the approach itself. A meta-analysis by Hew et al. [22] found favourable results for FC in general. Other studies did not find significant differences when compared to traditional learning sessions. The study conducted by Kaplan et al. [23] did not reveal any significant differences in the performance of basic clinical skills. Similarly, Uchida et al. [24] failed to demonstrate any statistically significant differences when teaching deep tendon reflexes.

Several studies have demonstrated the application of a FC approach in resuscitation training [17, 23, 24]. FC can bridge the gap by transferring knowledge of guidelines and pathways into self-directed learning. The transfer of non-technical skills, such as team skills, communication, or leadership, into pre-course learning time could preserve more in-class learning time for application and training. It is currently unknown whether FC is can achieve this.

### Aim

The objective of this study is to determine whether the flipped classroom approach, which includes an interactive, learner-paced learning session with audio-visual content, can enhance team performance and CPR quality. This will be measured by scores in team-based assessments and metrical performance data in a simulated resuscitation scenario.

## Methods

### Ethical approval

The study was approved by the Ethics Committee of the Chamber of Physicians at Westfalen-Lippe and the University of Münster (ID 2017–512-f-S) and registered at the German Clinical Trials Register (https://drks.de/search/de/trial/DRKS00013096), primary registration on 2017–10-04.

### Participants

In October 2017, all 118 final-year medical students enrolled in the Emergency Medicine course provided by the Department of Anaesthesiology, Intensive Care and Pain Medicine at the University of Münster were invited to participate in this study. As the course and its training were part of the obligatory curriculum, we presumed every participant to be eligible for inclusion. The instructors were residents and specialists in anaesthesia and emergency medicine. All instructors had experience in ALS training and received an additional one-hour introduction to the course and trial. All participants gave informed consent before the study and were randomised into teams.

**Fig. 1:**
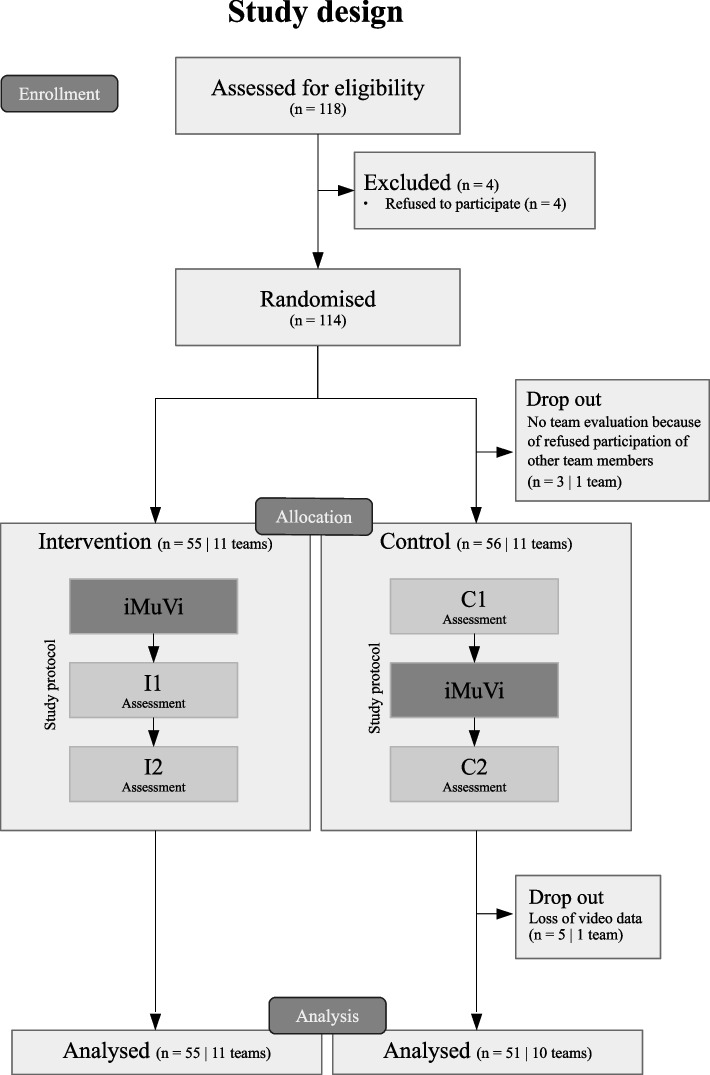
Modified Consolidated Standards of Reporting Trails (CONSORT) [25] flow diagram with study design: Subjects were randomised in teams of five. Control group (CG) and intervention group (IG) passed each one e-learning station (iMuVi) and two learning and assessment scenarios (indicated by numbers 1, 2) in a different order (C1: CG scenario 1, C2: CG scenario 2, I1: IG scenario 1, I2: IG scenario 2)

Fig. 1 provides a modified CONSORT flow diagram [25].

### Study design

This study was a cluster-randomised [26] controlled rater-blinded simulation trial. Fig. 1 illustrates the two-arm design. Participants received paper-based notes with identification numbers and a small computer script assigned participant numbers to teams of five. Each team was randomly assigned to either the control or the intervention group. The group assignment was neither open labeled nor were participants or trainers blinded.

Both the intervention and control groups underwent the same training stations, but in a different order. The intervention group commenced with the use of iMuVi (intervention, see below), followed by two separate ALS scenarios. The control group initially participated in an ALS simulation scenario, then used iMuVi, and finally performed a second ALS scenario. The scenarios involved in-hospital cardiac arrests with either ventricular fibrillation or asystole as the primary rhythm. Each simulation was followed by feedback rounds.

The study aimed to observe the impact of e-learning, hands-on training, or both on various parameters. Changes in parameters after using iMuVi were interpreted as the effect of e-learning, while changes independent of the use of iMuVi were interpreted as the effect of hands-on training.

The number of recruitable participants was limited by the curricular design of the faculty, which in turn limited the sample size.

### Intervention

**Fig. 2:**
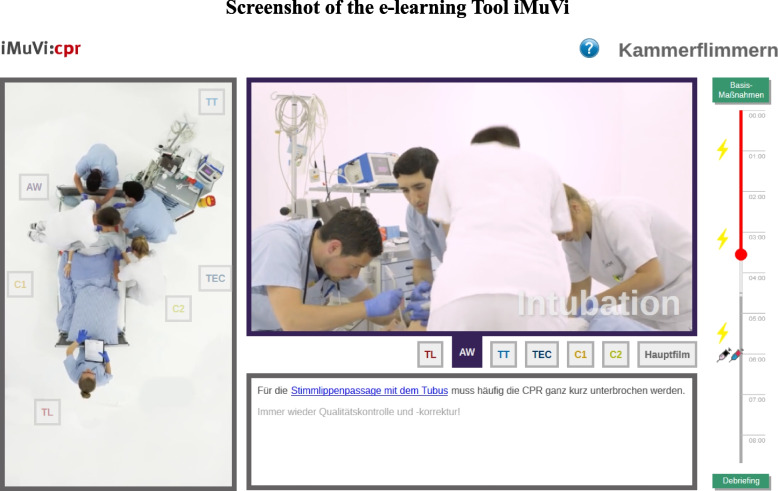
The left vertical frame shows the resuscitation process from above. The right movie frame provides one of seven perspectives on the scenario. The user can instantly switch between the perspectives using either the buttons connected to the team members in the vertical frame or the control panel below the vertical frame. The text frame beneath contains short textual information and linked supplemental information. The enhanced timeline enables the user to control the videos

The intervention comprised a 45-min e-learning session that utilized an interactive online course on team roles in resuscitation teams, known as iMuVi (Fig. 2).

The web-based Interactive Multi-perspective Video-e-Learning (iMuVi) [27] framework is designed to break down complex, time-sensitive, synchronous tasks into smaller learning chunks. The iMuVi framework can be used with interchangeable content. For example, the content could be a trauma room, an emergency room or even induction of general anaesthesia. In this trial we used resuscitation.

iMuVi provides text, audio and video content. Maximum control over the amount and speed of information delivery is the pedagogical paramount [28]. The central content was a video of a resuscitation scenario. The video provides up to seven perspectives of the same scene. Each perspective focuses on the tasks of one team member (role). Users can instantly change the perspective, control the playback of the videos and gather additional visual or textual information at any time, depending on their personal needs, prior knowledge and cognitive capacity (Fig. 2).

The video was produced by using seven independent digital video cameras synchronously. The fully scripted storyline concerns an elderly male patient who suffers a cardiac arrest on a regular ward. The attending nurse makes an emergency call and initiates basic life support. The emergency medical team is assembled and briefed ad hoc on the way to the patient. Particular attention is paid to closed-loop communication. The team complements the ongoing basic life support initiated by the ward nurses and provides defibrillation, airway management, i.v. access and other advanced interventions.

iMuVi has two main objectives. First: To provide very detailed content on medical knowledge, skills, communication principles and non-technical skills. Second, to act as a standardised model for emergency procedures and team collaboration. As implicit coordination is less time-consuming than explicit coordination, iMuVi is designed to provide a common concept of cooperation through a shared mental model [29].

### Outcome measures

As team performance was the primary outcome measure, all ALS scenarios were video and audio recorded. Team performance was assessed using a validated assessment tool: the Team Emergency Assessment Measure (TEAM) [30]. A multidisciplinary panel consisting of three faculty members from the Department of Anaesthesiology and one faculty member from the subspecialty of Emergency Medicine rated the recorded scenarios. The raters were blinded to group randomisation. To increase inter-rater reliability, the raters viewed four randomly selected recordings of the ALS scenarios together. An interactive workshop was held in which each item was discussed point by point and individual raters' scores were compared [31]. The raters then rated 19 to 20 videos each.

The secondary outcome measure was metric resuscitation performance. For this, four simulators (all Resusci Anne Simulator, Laerdal Medical Limited, Stavanger, Norway) recorded each chest compression, ventilation and defibrillation attempt.

### Data processing and statistical analysis

Statistical analysis was performed according to the level of measurement described below. *P* values < 0.05 were considered significant. All analyses were performed using (IBM SPSS Statistics, Version 25.0. Armonk, IBM Corp).

When data were normally distributed, t-tests were used to compare groups; otherwise, Mann–Whitney U tests were used. Associations between categorical variables were tested using chi-squared tests. Team performance and metric data were analysed using generalised estimating equations (GEE). In general, continuous variables are expressed as mean ± standard deviation (SD) or median [interquartile range], and categorical variables are expressed as numbers and percentages.

### Team performance

Team performance was measured using the TEAM checklist [30]. TEAM classifies eleven individual items into three categories (leadership, teamwork and task management). Each item is scored on a 5-point Likert scale. As suggested by the developers of TEAM, the TEAM category scores (T_lead_, T_work_, T_task_) were determined by calculating the mean of all individual items in each category. T_score_ was defined as the mean of all eleven individual items. T_mark_ is the global rating of TEAM on a 10-point scale.

Inter-rater reliability was statistically analysed. If the mean standard deviation of an item group was 1.5 or greater, a third reviewer was asked to rate these videos. The individual rating was defined as the median rating of all raters.

### CPR Metrics

CPR quality indicators were calculated using the recorded raw data from the CPR manikins. Compression artefacts were excluded based on the matching video information. Chest compression rate per minute (CC_Rate_) was calculated by taking the reciprocal of the compression-to-compression interval, excluding intervals greater than or equal to 2000 ms [32]. Chest compression depth (CC_Depth_) was available for each compression in the manikin data. Ratios of sufficient chest compression rate (100—120 min^−1^) and depth (RSCC_Rate_, RSCC_Depth_) and chest compression fraction (CC_Frac_) were calculated. Mean no flow time (mNFT) was the mean duration of all periods excluding chest compressions longer than 2000 ms.

### Generalised estimating equations (GEE) and data interpretation

CPR metrics and team performance were assessed in each of the two scenarios during the study. We used generalised estimating equations (GEE) with a linear model, normal distribution, identity link function and exchangeable correlation structure to test the study hypotheses regarding the influence of hands-on training or e-learning or both. Means reported using the GEE model are estimated marginal means (EMM).

Comparisons between scenarios and study arms were made to identify differences between scenario 1 and scenario 2 or intervention and control groups. Data are presented as pairs (scenario 1 vs scenario 2 and intervention vs control, respectively) with standard error and *P* value of a type III model test.

Pairwise differences were calculated for each individual assessment using a scenario*study group pairwise comparison. *P*-values were corrected using the Sequential Bonferroni method.

Results are presented using 95% Wald confidence intervals to indicate the precision of the estimated marginal means.

### Effect sizes

Effect sizes for the summative scoring parameter (T_score_, T_mark_) were calculated from the raw data and corrected for small sample size using Hedges’ g* algorithm [33] by comparing the team performance ratings of the control and intervention groups during scenario 1. Hegdes’ g* corrects for a small approximation error in the Hedges’ g function that occurs when the number of cases is small. Mean ± standard deviation (SD), two-tailed *P* value for the test of differences between means, 95% confidence interval of the difference between means, and Hedges’ g* are reported.

### Validity assumptions

Referring to the approach of a validation process proposed by Cook [34], based on Kane [35] and Messink [36], we assume that.

1. TEAM score reflects effective team performance, as
  - using a validated [30] and robust [37] measure
  - used by experienced and trained raters.

We further assume that

2) individual ratings are generalisable as
  - the TEAM score has been tested for internal reliability, and
  - inter-rater reliability has been tested and outliners have been rated by a third rater (see *Data Processing and Statistical Analysis* > *Team*).

Finally, we assume that

3) higher team scores reflect *better* performance because
  - the TEAM score has been developed on the basis of evidence of *good* team performance, and
  - team performance is a determinant of resuscitation quality in simulation [38, 39] and in real life defined as survival with good neurological recovery [9, 40], and
  - *better* team performance is reflected in *better* resuscitation metrics, defined as less no-flow time, more guideline-concordant compression rate and depth.

## Results

### Baseline characteristics

118 participants were invited to participate, but four declined. The remaining 114 participants were divided into 23 teams for the study. One team (CG) had to be excluded because of non-participating team members. Video data from one scenario in the control group was lost due to technical problems and these participants were excluded from the analysis. Data of 21 teams (IG: 10, CG: 11) and 106 participants (IG: 55, CG: 51) were available for performance analysis. Baseline characteristics for three participants and the metric data of one scenario were not available for analysis. These participants were not excluded from further analysis.

The mean age was 25.5 ± 3.8 years. 70 participants were female (68%). 11 participants (11%) reported having received medical training prior to their medical studies. 20 participants (19%) reported having participated in a real-life resuscitation attempt prior to the study. Other demographic data are shown in Table 1. There was no significant difference between the control and intervention groups on these items.

**Table 1:** Baseline characteristics of the study population. SD is standard deviation

Epidemiological data								
			*n*	%		mean	SD	*p*
Age (years)		Control group	51	50%		25.5	4.139	n.s.
		Intervention group	50^a^	50%		25.44	3.552	
Sex	female	Control group	34	49%	68%			n.s.
		Intervention group	36	51%				
	male	Control group	17	52%	32%			
		Intervention group	16	48%				
professional training prior medical studies	yes	Control group	4	36%	11%			n.s.
		Intervention group	7	64%				
	no	Control group	47	51%	89%			
		Intervention group	45	49%				
participation on real-life resuscitation	yes	Control group	7	35%	19%			n.s.
		Intervention group	13	65%				
	no	Control group	44	53%	81%			
		Intervention group	39	47%				

^a^ missing data, two subjects refused to give their age

## Outcomes

### Intervention vs. Control Group

There was no significant difference in baseline characteristics between then control and intervention groups (Tables 1 and 2) presents the metric and team performance data from this analysis.

**Table 2:** Metric data of chest compressions and team performance data: Statistics and Type III Tests of Model Effects of the inter-scenario comparison and the inter-arm comparison. Given values are estimated marginal mean (EEM). Std. Err indicates the standard error

Inter-scenario and inter-arm comparison of estimated marginal means											
		**Inter-scenario comparison**					**Inter-arm comparison**				
		Scenario 1		Scenario 2			Control		Intervention		
		Mean	Std. Err	Mean	Std. Err	p	Mean	Std. Err	Mean	Std. Err	*p*
CPR Metrics	Mean chest compression rate (CC_Rate_) [min^−1^]	118.3	2.4	112.8	1.9	n.s	116.2	2.3	114.9	2.2	n.s.
	Mean chest compression depth (CC_Depth_) [mm]	42.1	1.7	46.0	1.8	n.s	43.9	1.1	44.3	1.2	n.s.
	Ratio of sufficient chest compressions rate (RSCC_Rate_)	42.6	4.6	60.0	3.2	< 0.01	52.2	4.3	50.4	3.7	n.s.
	Ratio of sufficient chest compressions depth (RSCC_Depth_)	21.5	4.5	27.8	5.0	n.s	25.1	4.8	24.2	3.7	n.s.
	Mean chest compression fraction (CC_Frac_)	82.4	1.9	85.9	1.0	< 0.05	82.5	2.0	85.8	1.5	n.s.
	mean no flow time (mNFT) [sec]	6.4	0.6	5.2	0.4	n.s	5.7	0.6	6.0	0.7	n.s.
TEAM	Leadership (T_lead_)	4.0	0.1	4.4	0.1	< 0.05	3.9	0.1	4.5	0.1	< 0.001
	Teamwork (T_work_)	4.2	0.1	4.5	0.1	< 0.05	4.2	0.1	4.4	0.1	n.s.
	Task management (T_task_)	3.9	0.1	4.2	0.1	< 0.05	3.8	0.1	4.4	0.1	< 0.001
	Score (T_score_)	4.1	0.1	4.4	0.1	< 0.05	4.1	0.1	4.4	0.1	< 0.01
	Global Rating (T_mark_)	6.6	0.3	7.3	0.2	< 0.05	6.4	0.3	7.4	0.2	< 0.01

### Team performance

Except for T_work_, all TEAM scores were higher in the intervention group compared to the control group. EMM of global TEAM rating mark (T_mark_) was 7.4 (95% CI 6.9 to 7.8) vs 6.4 (95% CI 5.8 to 6.3 (*P* < 0.01), leadership (T_lead_) 4.5 (95% CI 4.3 to 4.6) vs 3.9 (95% CI 3.6 to 4.1) (*P* < 0.001), Task (T_task_) 4.4 (95% CI 4.2 to 4.5) vs 3.8 (95% CI 3.5 to 4.0) (*P* < 0.001), and summative score (T_score_) 4.4 (95% CI 4.3 to 4.5) vs 4.1 (95% CI 3.9 to 4.3) (*P* < 0.01).

### CPR Metrics

There was no significant difference in any CPR parameter between control and intervention in this comparison.

### Effect sizes

Comparison of mean scores between the control and intervention groups showed significantly higher scores for the intervention group and thus large effect sizes: T_score_ was 4.4 ± 0.4, respectively 3.8 ± 0.5 (*P* < 0.01, Hedges' g* = 1.4). T_mark_ ratings: 7.5 ± 1.3, respectively 5.6 ± 1.3 (*P* < 0.01, Hedges' g* = 1.4).

### Scenario 1 vs. Scenario 2

The inter-scenario comparison compares the performance data of the first and second scenarios for all teams, irrespective of their randomisation status.

### Team performance

EMM of all TEAM scores were significantly greater in scenario 2 then in scenario 1.

### CPR Metrics

Teams in the first scenario performed 42.6% (95% CI 33.6 to 51.5) of compressions at the correct rate (RSCC_Rate_), teams in the second scenario performed 60.0% [95% CI 53.6 to 66.3] correctly. The difference was significant (*P* < 0.01).

The chest compression fraction (CC_Frac_) was lower in the first scenario at 82.4% (95% CI 78.5 to 86.3) compared to the second scenario and at 85.9% (95% CI 84 to 87.8) (*P* < 0.05) respectively.

#### Inter-assessment comparison

 Inter-assessment comparison compares the performance data of each individual assessment (scenario), i.e. scenario 1 versus scenario 2 and control versus intervention. Type III model testing on EMM was performed to identify differences between each individual assessment.

### Team performance

**Fig. 3:**
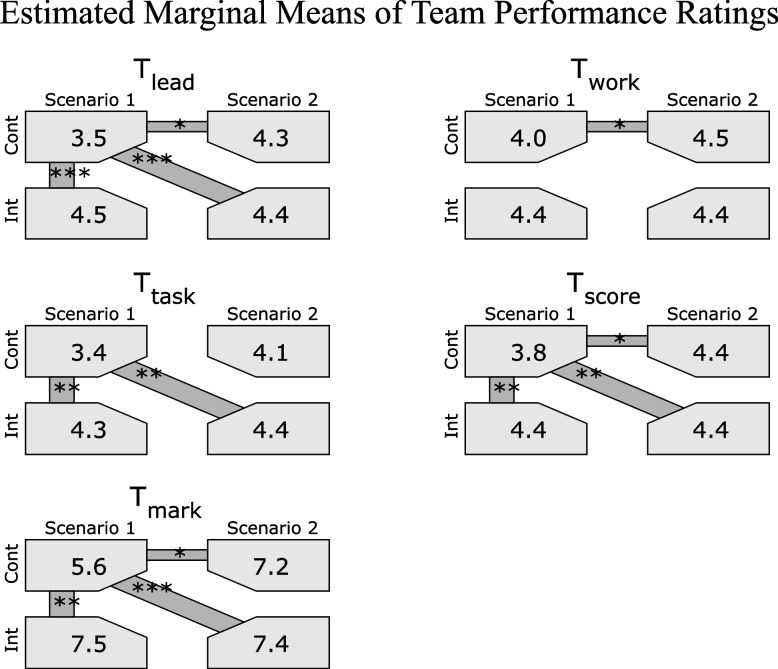
Estimated marginal means of tea performance ratings for each rating category separated by assessment (light grey). Signifikant differenes between the assessments are marked with bars (dark grey). *P* values of pairwise comparison corrected with Sequential Bonferroni. Significant levels: *: < 0.05; **: < 0.01; *** < 0.001

Type III model testing on EMM of all TEAM parameters were significant for scenario*study-group interaction (see Fig. 3, Table 3): T_mark_ (*P* < 0.05), T_lead_ (*P* < 0.01), T_work_ (*P* < 0.05), T_task_ (*P* < 0.05), T_Score_ (*P* < 0.05).

**Table 3:** Team performance data: Pairwise comparisons of EMM between each single assessment. Given values are EEM. Std. Err indicates the standard error. Arm*Scene indicates *p*-values of Type III Tests of Model Effect. Inter-assessment Comparison

Inter-assessment comparison										
		**Inter-assessment comparison**								
		Scenario 1				Scenario 2				
		Control		Intervention		Control		Intervention		Arm*Scene
		Mean	Std. Err	Mean	Std. Err	Mean	Std. Err	Mean	Std. Err	*p*
TEAM	Leadership (T_lead_)	**3.5**	0.2	**4.5**	0.1	**4.3**	0.2	**4.4**	0.1	< 0.01
	Teamwork (T_work_)	**4.0**	0.1	**4.4**	0.1	**4.5**	0.1	**4.4**	0.1	< 0.05
	Task management (T_task_)	**3.4**	0.2	**4.3**	0.1	**4.1**	0.2	**4.4**	0.1	< 0.05
	Score (T_score_)	**3.8**	0.2	**4.4**	0.1	**4.4**	0.1	**4.4**	0.1	< 0.05
	Global Rating (T_mark_)	**5.6**	0.4	**7.5**	0.4	**7.2**	0.4	**7.4**	0.2	< 0.05

### CPR Metrics

EMM of mNFT was 6.4 (95% CI 5.1 to 7.7) in the first scenario and 4.9 (95% CI 3.7 to 6.1) in the second scenario of the control group (*P* < 0.05).

## Discussion

The prevention and treatment of cardiac arrest, a major cause of death, is an important social mission [1, 2, 41]. Different targets are addressed by prevention strategies. The development and implementation of educational approaches for therapy is complex, as cardiac arrest is fortunately a rare event, but is associated with considerable time pressure, high workloads, high personal demands, which require effective training, but are met with limited resources. High-quality resuscitation requires knowledge [38], technical [42] and non-technical skills [38, 43]. Acquiring these skills takes time. Shifting the acquisition of knowledge to a phase prior to the classroom course is a reliable principle that allows for reflection on what has been learned within the classroom setting [15], thus allowing for more efficient hands-on time. Shifting the acquisition of non-technical skills such as team leadership was the aim of iMuVi. This study was designed to test the effectiveness of the FC approach. The findings are consistent with a recent study by Hassan [44]. Their study found evidence of improved CPR performance for a group that watched a video instead of a live demonstration before hands-on training.

### Team performance

Team performance was assessed by reviewing videotapes recorded by physicians experienced in the field of resuscitation, using the Team Emergency Assessment Measure (TEAM) [32] developed by Cooper et al., in accordance with instrument development guidelines. The authors carefully conceptualised the content, analysed the performance domains and performed appropriate statistical testing to validate their assessment instrument. The raters in our study were previously trained on this rating instrument, as suggested by Subkoviak [33]. At least two experts rated each scenario. A third assessor assessed scenarios with inconsistent ratings. Referring to Cook’s [34] synopsis of the evidence validity frameworks by Messick [36] and Kane [35], we assume that the content and the internal structure of team performance outcomes are sufficient.

As hypothesised, our data indicate that flipped classroom learning can improve team performance, supported by a significant and large effect size increase in global rating (T_mark_) score and combined overall performance score (T_score_). As Cooper and colleagues found in their observations of resuscitation teams in real-life situations, team structure is highly correlated with dynamism and accuracy of interventions during resuscitation [9]. Experimental studies have also shown that team structure and leadership influence performance [10] and that pre-formed teams have less hands-off time [11]. Based on the existing literature and our findings, we found surrogate parameters that the use of iMuVi led to better overall resuscitation performance.

While most of the studies reviewed by O’Dea [45] used simulation to improve team performance, a few used didactic teaching alone. The latter showed smaller effect sizes than simulation training. McEwan analysed moderator variables of learning outcomes in team training. More theoretical approaches such as lectures and presentations had little or no impact on learning [46]. In contrast to the above literature, we found large effect sizes for a more cognitive approach to learning. We used an existing tool, iMuVi, for pre-course learning. The strength of iMuVi lies in its ability to decompose a time-critical complex procedure into small learning chunks. Unlike lectures and presentations, the e-learning tool we used encourages *interactive* engagement with the content. Interaction is a crucial aspect of e-learning [44, 47]. The tool is user-friendly and has been shown to achieve high scores [48] on System Usability Scale [49]. It allowed for control of the time line and a learner-paced density of information, referred to as fostering learning [50–55]. Thus, the quality and presentation of pre-course learning content will have a significant impact on learning outcome. Course developers should take this into account if they flip the classroom, e. g. to promote pre-course teamwork learning.

### CPR Metrics

Metric CPR parameters were assessed using the simulation manikin’s internal recording. The manikin software calculates metric statistics of chest compressions. These statistics are prone to errors as manipulation of the manikin could be interpreted as chest compressions. Therefore metrics were calculated from raw data using video-based plausibility checks. Time-related metrics such as compression rate (CC_Rate_) or chest compression fraction (CC_Frac_) and derived parameters were available with good reliability. Compression depth is highly contingent upon the technological solution employed for measurement. We used the manikin’s own internal solution. As mechanical parts wear, measurements will change over time and may differ between simulators. All simulators used underwent a simultaneous general overhaul prior to the study, and the teams used the same simulator in both scenarios. However, compression depth data are less reliable than time-related data. We assume that relative changes are more reliable than absolute compression depth (in cm).

iMuVi was not designed to improve metric parameters of resuscitation. As expected [56] e-learning had no relevant impact on most CPR parameters. Only chest compression fraction (CC_Frac_) and ratio of sufficient chest compression rate (RSCC_Rate_) improved from scenario 1 to scenario 2 independently of the use of iMuVi. These metrics appear to be influenced by physical training in simulation rather than e-learning. Training with live feedback has been shown to be effective in training [57], and the use of live feedback in real-life resuscitations is part of the ILCOR resuscitation guideline [58]. Curricular training should incorporate iMuVi and live feedback to further improve the quality of CPR.

Only the mean no-flow time was significantly better in the control group after the use of iMuVi and did not improve in the intervention group. In contrast to the metric parameters mentioned above, this parameter is more dependent on team coordination than on motor skills. The consistency between metric results and the team performance ratings supports our interpretation of the team performance ratings that the use of iMuVi improved resuscitation performance.

### Generalisability

We found evidence that iMuVi as a tool in a flipped-classroom approach was effective in improving resuscitation performance in our study cohort. Whether this applies to students outside of this study or to other tools in FC approaches, needs to be discussed.

Effectiveness will depend on the tool and the learners. The participants in our study were in their final year of medical school. They had extensive medical knowledge and experience from several basic life support courses, which may have influenced the result. They had dedicated learning time for iMuVi. This should be true of an ideal flipped classroom outside of a trial as well, but exclusivity may be compromised in a less controlled environment. We cannot say for sure whether iMuVi will be as effective outside a trial. As the intervention showed large effect sizes on team performance in our trial, we expect that the flipped classroom approach to have some effect, but probably with smaller effect sizes. A follow-up might provide evidence.

iMuVi was developed on the basis of intensive literature studies on content and structure. The importance of correct presentation of medical content is obvious. The importance of a good structural approach may be less obvious, but equally important. Control over the amount and speed of content delivery is paramount [28, 47, 55]. Mayer and colleagues [55] highlighted that learning outcomes are influenced by the information delivered and the order of complexity of information presented. Referring to cognitive load theory, they conclude that learning should start with the presentation of small chunks of a concept and end with the view of the whole process. We assume that iMuVi has implemented these principals. In terms of generalisability, other FC approaches need to take these pedagogical principals into account in order to achieve similar results.

We used a resuscitation attempt as a paradigmatic model of team collaboration in time-critical events. This study did not gather evidence on the transferability of our findings to procedures other than cardiac arrest. Further studies should investigate whether a FC approach is suitable for team training for routine procedures others than resuscitation.

### Limitations

Simulator studies are widely used to obtain evidence. In some cases, they are the only ethical way to generate knowledge. Despite their merits, they have limitations. Even if simulation closely resembles real-life produces comparable stress levels, it is unclear how much stronger learning effects are in real-life resuscitation attempts [59]. We used a validated measure of team performance and trained assessors. As these scores are based on the subjective perception of the raters, the data should be interpreted with caution, although inter-rater reliability was adequate in this study [31].

The participants were final year medical students with little clinical experience, all recruited from one university. In addition to training, resuscitation performance depends on real-life experience. In a report by Thorne et al*.* clinical experience rather than time spent on an electronic ALS course was identified as an independent predictor of course success [60]. Neither courses nor tools such as iMuVi can replace clinical experience.

This study may overestimate the effect outside of a trial setting. Students had planned exclusive time to use iMuVi. The learning outcome will be lower if the learning time conflicts with other activities.

## Conclusion

The use of iMuVi, a learner-paced e-learning flipped classroom approach, can improve team performance in resuscitation training and should be used to prepare team members and leaders prior to attending hands-on training.

## Data Availability

iMuVi is available in German for the purpose of demonstration on https://imuvi.uni-muenster.de/ with login *bmc* and password *education.* The access may be withdrawn without further notice. Contact: Hendrik Ohlenburg, ohlenburg@uni-muenster.de

## Abbreviations

CC_Depth_: Chest compression depth (in cm)
CC_Frac_: Chest compression fraction
CC_Rate_: Chest compression rate per minute
CI: Confidence interval
EMM: Estimated marginal means
mNFT: Mean no flow time
RSCC_Depth_: Ratio of sufficient chest compression depth
RSCC_Rate_: Ratio of sufficient chest compression rate
SD: Standard deviation
